# A knowledge‐based planning model to identify fraction‐reduction opportunities in brain stereotactic radiotherapy

**DOI:** 10.1002/acm2.70055

**Published:** 2025-02-21

**Authors:** Shane McCarthy, William St. Clair, Damodar Pokhrel

**Affiliations:** ^1^ Medical Physics Graduate Program Department of Radiation Medicine University of Kentucky Lexington Kentucky USA

**Keywords:** clinical efficiency, HyperArc, multiple brain lesions, patient time in the clinic, planning automation, RapidPlan, SIML, stereotactic radiotherapy, treatment planning

## Abstract

**Objective:**

To develop and validate a HyperArc‐based RapidPlan (HARP) model for three‐fraction brain stereotactic radiotherapy (SRT) plans to then use to replan previously treated five‐fraction SRT plans. Demonstrating the possibility of reducing the number of fractions while achieving acceptable organs‐at‐risk (OAR) doses with improved target biological effective dose (BED) to brain lesions.

**Methods:**

Thirty‐nine high‐quality clinical three‐fraction HyperArc brain SRT plans (24–27 Gy) were used to train the HARP model, with a separate 10 plans used to validate its effectiveness. Fifty‐eight five‐fraction HyperArc brain SRT plans (30–40 Gy) attempted to be retrospectively replanned for three fractions scheme using the HARP model. All planning was done within the Eclipse treatment planning system for a TrueBeam LINAC with a 6 MV‐FFF beam and Millenium 120 MLCs and dosimetric parameters were analyzed per brain SRT protocol.

**Results:**

The HyperArc RapidPlan model was successfully trained and tested, with the validation set demonstrating a statistically significant (*p* = 0.01) increase in GTV D_100%_ from 28.5 ± 0.7 Gy to 29.4 ± 0.6 Gy from the original to RapidPlan plans. No statistically significant differences were found for the OAR metrics (*p* > 0.05). The five‐fraction replans were successful for 20 of the 58 five‐fraction brain SRT plans. For those 20 successful brain SRT plans, the maximum doses to OAR were clinically acceptable with a three‐fraction scheme including an average V_18Gy_ to Brain‐PTV of 9.9 ± 5.9 cc. Additionally, the replanned five‐fraction brain SRT plans achieved a higher BED to the tumors, increasing from a GTV D_100%_ of 52.9 ± 4.5 Gy for the original five‐fraction plans to 57.3 ± 3.1 Gy for the three‐fraction RapidPlan plans. All RapidPlan plans were generated automatically, without manual input, in under 20 min.

**Conclusions:**

The HARP model developed in this research was used to successfully identify select five‐fraction plans that were able to be reduced to three‐fraction SRT treatments while achieving clinically acceptable OAR doses and improved target BED. This tool encourages a fast and standardized way to provide physicians with more options when choosing the necessary fractionation scheme(s) for HyperArc SRT to single‐ and multiple brain lesions.

## 1 INTRODUCTION

Brain metastases affect 20% to 30% of patients with systemic cancer, outnumbering all other intracranial tumors combined and continuing to increase^1^, ^2^ Depending on the tumor size, location, and number of metastases, the chosen treatment course can be surgical resection, whole brain radiotherapy (WBRT), or stereotactic radiotherapy and radiosurgery (SRT/SRS).^3^, ^4^, ^5^ As technology evolves, leading to improved accuracy and effectiveness of SRS/SRT, more and more physicians are pursuing the avenue of SRS/SRT for larger numbers of metastases and larger tumor sizes.^6^, ^7^, ^8^

As more complex treatment plans are selected for SRS/SRT, the necessary time, effort, and expertise to generate clinically acceptable plans is increased. This limits the accessibility of convenient, high‐quality radiation treatment to academic centers and well‐resourced community clinics with experienced planners. Recent tools, like Varian's HyperArc and RapidPlan, have helped bridge the gap by facilitating an increase in the ease of creation of high‐quality, clinically acceptable SRS/SRT treatments for brain lesions.^9^, ^10^, ^11^, ^12^, ^13^

When considering SRS (single fraction) or SRT (multi‐fraction), the dose spread to the normal brain is used for assessing brain toxicity and radiation‐induced brain necrosis, as demonstrated in numerous studies investigating brain toxicity for brain SRT.^4^, ^5^, ^6^, ^14^, ^15^ As the number and size of metastases increase, physicians may opt to limit toxicity by increasing the number of fractions. Additionally, if a metastasis is in close proximity to an organ‐at‐risk (OAR), the physician might decide on more fractions. Increasing the number of fractions utilizes the inherent radiobiological benefits that come with fractionation to better respect OAR doses while still delivering an acceptable, though slightly less biologically effective dose (BED), to the tumor.

Herein, a HyperArc‐based RapidPlan (HARP) model is trained and tested on high‐quality clinically used three‐fraction HyperArc SRT plans and then used to replan a previously delivered five‐fraction HyperArc SRT plan to a three‐fraction prescription. An objective planner such as RapidPlan can allow clinics to create consistent, high‐quality radiation plans. This provides attending physicians the opportunity to achieve an acceptable normal brain dose in three fractions for cases where five fractions were initially thought necessary. This would reduce patient time in the clinic, improving patient comfort and compliance while delivering a biologically more effective dose to the tumors.

## 2 METHODS AND MATERIALS

### 2.1 Patient selection

In total, 107 previously treated, high‐quality HyperArc SRT single‐isocenter multi‐ and single‐lesion plans were used throughout this research after obtaining Institutional Review Board approval from our institute. All patients had the gross tumor volume (GTV) delineated on contrast‐enhanced high‐resolution MRI images by the physician. A 2 mm expansion to the GTV was applied to generate the planning target volume (PTV) for most tumors, with smaller and/or asymmetrical margins being used for special cases. The original, clinically delivered SRT plans were planned for and delivered every other day on a Varian TrueBeam LINAC with a 6 MV‐FFF beam using the HyperArc module with the Millenium 120 multi‐leaf collimator (MLC). Treatment planning was done in Eclipse v15.6 and Eclipse v16.1. Arc selection, collimator angle, and isocenter placement were automated within the HyperArc module and then manually refined by an experienced HyperArc planner if necessary. All plans were manually generated by an expert HyperArc planner (approximately 2 –4 hours), approved by the attending physicians, and passed all physics second checks, including patient‐specific pretreatment quality assurance through EPID‐based portal dosimetry and an in‐house independent Monte Carlo‐based recalculation of dose.^16^, ^17^

Forty‐nine plans were three‐fraction brain SRT plans used to generate and validate the HARP model. The remaining fifty (58) plans were five‐fraction SRT plans used to demonstrate the ability of the HARP model to reduce the number of fractions from five to three.

### 2.2 Three‐fraction model creation

The three‐fraction SRT plans consisted of a total of 102 metastases across 49 plans with total dose prescriptions of 24 Gy (4) and 27 Gy (45). There were 29 metastases in the frontal lobe, 19 in the parietal lobe, 21 in the occipital lobe, 8 in the temporal lobe, and 25 in the cerebellum. For model creation, the plans were split into a training and testing set based on the size, location, and number of lesions. Both the training and testing sets were constructed to represent the overall population as best as possible. The training set resulted in 85 total lesions with an average of 2.2 ± 1.6 lesions per patient and an average PTV volume of 4.89 ± 5.94 cc. The testing set had 17 total lesions with an average lesions per patient of 1.7 ± 0.8 lesions. The average PTV volume for the testing set was 6.45 ± 7.57 cc. Table 1 outlines additional details of the data split for this cohort.

**TABLE 1:** Plan demographics for RapidPlan training and testing set.

		Training	Testing
Number of patients		39	10
Prescription dose	24 Gy	4	0
	27 Gy	35	10
Lesion location	Frontal (L/C/R)	8/2/14	1/2/2
	Parietal (L/C/R)	7/1/7	1/0/3
	Occipital (L/C/R)	5/3/10	1/0/2
	Temporal (L/C/R)	2/1/3	2/0/0
	Cerebellum (L/C/R)	11/1/10	2/0/1
Total number of lesions		85	17
Lesions per patient		2.2 ± 1.6 (1–8)	1.7 ± 0.8 (1–3)
Distance to isocenter (cm)		3.26 ± 2.45 (0.00–8.27)	3.64 ± 2.45 (0.04–8.20)
PTV volume (cc)		4.89 ± 5.94 (0.07–31.82)	6.45 ± 7.57 (0.39–27.31)
PTV diameter (cm)		2.38 ± 1.26 (0.67–7.45)	2.66 ± 1.23 (1.03–6.25)
Brain volume (cc)		1300 ± 107 (929–1544)	1363 ± 115 (1217–1584)

*Note*: When applicable, values are reported as mean ± standard deviation (range). Distance to the isocenter is measured from the isocenter to the center point of the tumor. Lesion location reported as left/center/right in the brain parenchyma.

Abbreviation: PTV, planning target volume.

#### 2.2.1 Model training

Training the HARP model consisted of extracting the brain SRT plan information to the model, running the DVH estimation algorithm, and then assessing the results of the training through the provided statistics that indicate potentially influential structures as well as geometric and dosimetric outliers. After analyzing the statistics, model refinement is performed by unmatching specific structures or entirely removing plans from the model. This is an iterative process that stops when no significant outliers are identified as being detrimental to the effectiveness of the model. Several studies have outlined this process in greater depth.^13^, ^18^, ^19^ The final optimization objectives can be found in the appendix (Table A1). For this model, the SRS NTO was used with a priority of 100.

#### 2.2.2 Model testing

To test the capabilities of the three‐fraction model, the 10 testing plans were retrospectively replanned using the model, and the resulting plans were compared to the original, clinically delivered plans. To make a more direct comparison between the HARP model and the lack thereof, identical plan geometry was used between the original manual plan and the automated RapidPlan plan. As previously mentioned, this geometry was initially established by the HyperArc module and then fine‐tuned by an experienced HyperArc planner. Following the duplication of the beam geometry, the remainder of the planning process was fully automated using Eclipse Scripting API (ESAPI), this includes the DVH estimation, optimization, dose calculation, and normalization, all within Eclipse v16.1 (Varian Medical Systems, Palo Alto, CA). The RapidPlan plans were normalized such that the total PTV D_95%_ received the same dose as received in the original manual plan.

#### 2.2.3 Model evaluation

Plans were evaluated for target coverage, such as the D_100%_ of the PTVs, D_100%,_ and D_0.03cc_ of the GTVs, as well as the Paddick conformity index and other metrics for the PTVs.^20^, ^21^ Furthermore, dose metrics were obtained for various OARs of interest, including the optic pathway, cochlea, eyes, brainstem, spinal cord, and skin, following Alliance A071801 criteria, AAPM TG 101 report, and in‐house clinical plan evaluation criteria.^22^, ^23^ Of particular interest was the dose spread to the normal brain tissue; this was quantified using V_18Gy_ as recommended by the Radiosurgery Society Guidelines.^24^

### 2.3 Five‐fraction replanning

The five‐fraction plans consisted of a total of 192 metastases across 58 brain SRT plans with total dose prescriptions ranging from 25 to 40 Gy. There were 52 lesions in the frontal lobe, 44 in the parietal lobe, 23 in the occipital lobe, 29 in the temporal lobe, 36 in the cerebellum lobe, and 8 located in other areas near the brain. On average, there were 3.3 lesions per patient with an average PTV volume of 9.06 cc. Additional demographics for the five‐fraction plans are presented in Table 2.

**TABLE 2:** Plan demographics for five‐fraction plans selected to be replanned to three‐fractions.

Number of patients		58
Prescription dose	25 Gy	5
	30 Gy	47
	35 Gy	7
	40 Gy	1
Lesion location	Frontal (L/C/R)	24/7/21
	Parietal (L/C/R)	19/7/18
	Occipital (L/C/R)	9/6/8
	Temporal (L/C/R)	10/5/14
	Cerebellum (L/C/R)	14/12/10
	Other	8
Total number of lesions		192
Lesions per patient		3.3 ± 3.4 (1–14)
Distance to isocenter (cm)		4.65 ± 2.10 (0.00–9.21)
PTV volume (cc)		9.06 ± 17.48 (0.06–99.43)
PTV diameter (cm)		2.67 ± 1.79 (0.63–9.58)
Brain volume (cc)		1369 ± 158 (1107–1732)

*Note*: When applicable, values are reported as mean ± standard deviation (range). Distance to the isocenter is measured from the isocenter to the center point of the tumor. Lesion location reported as left/center/right in the brain parenchyma.

Abbreviation: PTV, planning target volume.

#### 2.3.1 Planning technique

A new HyperArc plan was generated for each five‐fraction plan with a prescription of 27 Gy in three‐fractions on a TrueBeam LINAC with a 6 MV‐FFF beam and the Millenium 120 MLCs. The original PTVs and GTVs were selected as targets within the HyperArc module and the isocenter and beam selection was generated automatically. After creating the HyperArc plan, an in‐house ESAPI script was used to automate the DVH estimation, optimization, dose calculation, and normalization of the plans as mentioned before. Plans were normalized such that all PTVs received at least the prescription dose. Dose to medium reporting mode was used for the AcurosXB algorithm with a 1.25 mm calculation grid size in Eclipse v16.1.

#### 2.3.2 Plan evaluation and data analysis

The replanned five‐fraction plans were analyzed for target coverage through the PTV D_100%_ as well as the GTV D_100%_ and D_0.03cc_. Additionally, Paddick's conformity index, the RTOG conformity index, the homogeneity index, and the gradient index were all calculated for the PTVs. OAR doses were recorded for the brain, optic pathway, brainstem, spinal cord, skin, and cochlea, referring to current accepted criteria for three‐fraction SRT as well as in‐house clinical protocols.^22^, ^23^, ^24^ For the three‐fraction manual clinical brain SRT plans versus HARP model validation plans, the comparison of dosimetric parameters was performed using the Wilcoxon signed‐rank test (nonparametric) or paired samples *t*‐test (parametric) and a significance level of *p*‐value of < 0.05. For the five‐fraction replanned brain SRT plans, statistical analysis between the five‐fraction plans and the replanned three‐fraction plans is not meaningful due to the different dose prescriptions. In addition to the three‐fraction brain SRT protocol criteria, the mean values, standard deviations, and ranges are reported.

## 3 RESULTS

### 3.1 Three‐fraction model

The HARP model was successfully trained on all 39 of the three‐fraction training plans, with 507 OAR structures initially matched, 73 structures identified as outliers, and 26 structures removed from the model. The model was then validated on the 10 brain SRT testing plans. On average, the testing plans took 11.9 ± 2.9 min (6.3 – 16.6 min) to create, measured from the end of establishing beam geometry (duplicating that of the original plan) to the final dose being calculated. The DVH estimation took approximately 40 s on average, with the first round of optimization taking just under 6 min on average. The intermediate dose calculation, second round of optimization, and final dose calculation took approximately 2 min each. The RapidPlan plans resulted in an average of 2684 ± 274 MUs (2183–3134 MUs) compared to the original plans’ 2702 ± 780 MUs (2022–4859 MUs). The MU difference was statistically insignificant (*p* = 0.94).

#### 3.1.1 Target coverage

As previously mentioned, the SRT RapidPlan plans’ total PTV D_95%_ was normalized to that of the original clinical plans, as such, no PTV D_95%_ values are reported. The RapidPlan plans demonstrated a statistically significant (*p* = 0.01) increase in the D_100%_ of the GTVs with an average value of 29.4 ± 0.6 Gy compared to 28.5 ± 0.7 Gy of the manual plans. Additionally, a statistically significant (*p* = 0.02) decrease in the D_100%_ of the PTVs was observed, with the RapidPlan plans receiving an average of 24.3 ± 0.7 Gy compared to the manual plans’ 25.0 ± 0.7 Gy. All other reported metrics were found to be statistically comparable and are reported in Table 3.

**TABLE 3:** RapidPlan model testing results for target coverage and OAR doses across 10 test plans.

Structure	Metric	Criteria	Manual plan	RapidPlan	*p*‐value
PTV	D_100%_ (Gy)	≥24.3^a^	25.0 ± 0.7 (23.6–25.8)	24.3 ± 0.7 (23.1–25.5)	**0.02**
	Paddick CI	≥0.9^a^	0.90 ± 0.05 (0.73–0.95)	0.89 ± 0.04 (0.80–0.94)	0.13
	RTOG CI	1.0–1.5^a^	1.07 ± 0.08 (0.97–1.33)	1.07 ± 0.07 (0.98–1.22)	0.84
	HI	≥0.15^a^	0.15 ± 0.04 (0.09–0.22)	0.16 ± 0.03 (0.12–0.21)	0.26
	GI	3.0–4.0^a^	3.89 ± 1.38 (2.56–7.67)	3.52 ± 0.79 (2.66–5.39)	0.06
GTV	D_100%_ (Gy)	≥27^a^	28.5 ± 0.7 (27.2–29.9)	29.4 ± 0.6 (28.4–30.4)	**0.01**
	D_0.03cc_ (Gy)	<35.1^a^	31.7 ± 1.3 (29.5–33.5)	31.9 ± 0.8 (30.6–33.5)	0.66
Brain‐PTV	V_18Gy_ (cc)	<26^b^	8.81 ± 5.19 (4.03–19.36)	8.06 ± 4.49 (2.94–17.38)	0.12
Optic pathway	D_0.2cc_ (Gy)	<13.8^c^	1.6 ± 1.7 (0.1–5.3)	1.3 ± 1.1 (0.3–3.4)	0.18
	D_0.03cc_ (Gy)	<17.4^c^	2.0 ± 2.1 (0.1–7.1)	1.6 ± 1.4 (0.3–4.6)	0.29
Brainstem	D_0.5cc_ (Gy)	<18^c^	2.3 ± 1.6 (0.4–5.1)	2.6 ± 1.9 (0.6–6.9)	0.21
	D_0.03cc_ (Gy)	<23.1^c^	2.8 ± 1.9 (0.5–6.1)	3.2 ± 2.4 (0.8–8.8)	0.14
Spinal cord	D_0.35cc_ (Gy)	<18^d^	1.1 ± 1.4 (0.0–4.0)	0.9 ± 1.0 (0.0–2.8)	0.17
	D_0.03cc_ (Gy)	<21.9^d^	1.4 ± 1.7 (0.0–5.3)	1.1 ± 1.3 (0.0–3.9)	0.15
Skin	D_0.03cc_ (Gy)	<13.5^a^	10.4 ± 6.1 (4.6–26.6)	10.9 ± 6.8 (4.3–27.0)	0.53
Left cochlea	D_0.03cc_ (Gy)	<17.1^c^	2.2 ± 2.9 (0.2–10.0)	1.5 ± 1.5 (0.3–5.2)	0.27
Right cochlea	D_0.03cc_ (Gy)	<17.1^c^	1.2 ± 1.3 (0.1–4.5)	1.1 ± 1.5 (0.2–5.1)	0.42

*Note*: Values are reported as mean ± standard deviation (range). RapidPlan plans’ PTV Total of D_95%_ was normalized to the clinical plans’ PTV Total of D_95%_. Statistically significant values (*p* < 0.05) are bolded.

Abbreviations: GTV, gross tumor volume; OAR, organs‐at‐risk; PTV, planning target volume.

^a^ Clinical Protocol.

^b^ RSS Guidelines.^24^

^c^ Alliance A071801.^23^

^d^ AAPM Task Group 101.^22^

#### 3.1.2 Dose to OARs

**FIGURE 1:**
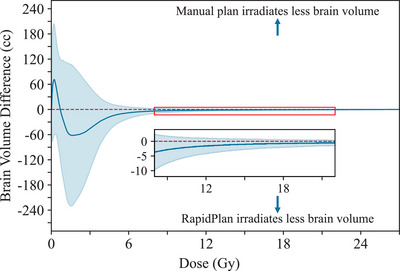
Normal brain volume difference between manual brain SRT plans and RapidPlan model as a function of dose. The blue solid line represents the average brain volume difference with shadowing outlining ± 1 standard deviation for all 10‐test brain SRT plans.

All reported OAR metrics were found to be statistically insignificant between the manual and RapidPlan SRT plans (Table 3). Although not statistically significant, one notable trend in the Brain‐PTV structure's V_18Gy_ was observed: a decrease in the average value from 8.81 cc for the manual plan to 8.06 cc for the RapidPlan plan, as well as a decrease in the maximum value from 19.36 cc for the manual plan and 17.38 cc for the RapidPlan plan. The difference in irradiated normal brain volume is highlighted in Figure 1.

#### 3.1.3 Example test plan

**FIGURE 2:**
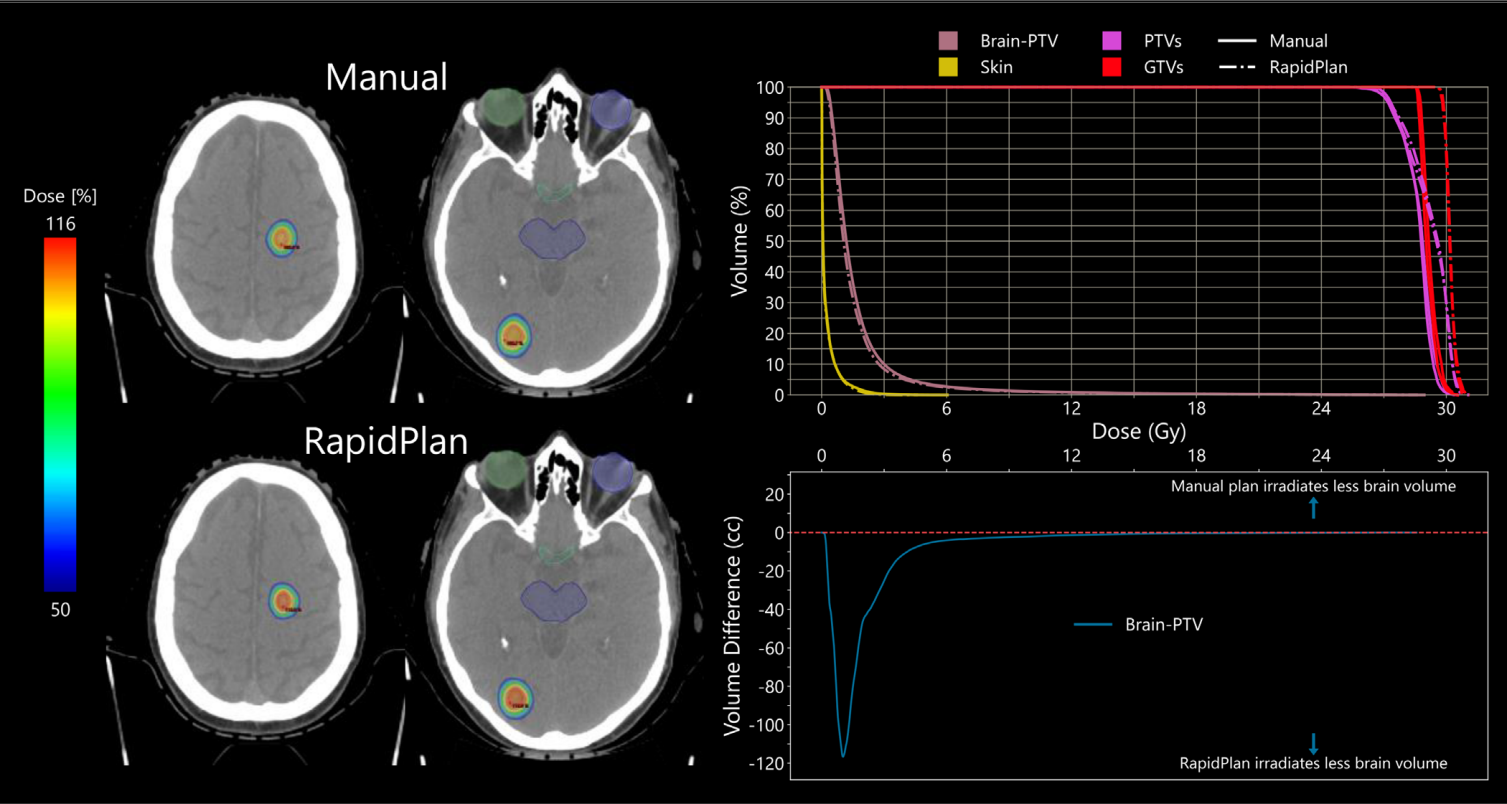
Isodose color wash distributions, DVHs, and normal brain volume difference graph for Test Patient #5 from the testing set of the three‐fraction HARP brain SRT model. This patient had two metastases (1.56 cc and 1.28 cc) prescribed 27 Gy in three fractions. Contours shown on the isodose distributions are the chiasm (green), brainstem (blue), eyes (green and blue), PTVs (pink), and GTVs (red). The graph in the bottom right is showing the difference in the Brain‐PTV volume receiving dose as a function of that dose.

Figure 2 shows the isodose distributions, DVHs, and normal brain volume difference graph for Testing Plan #5 of the 10 testing plans used to validate the model. This patient had two metastases, one located in the right occipital lobe with a PTV volume of 1.56 cc and one located in the left parietal lobe with a PTV volume of 1.28 cc. The patient was prescribed 27 Gy in three‐fractions. Due to the lesions’ distance from OARs, the normal brain toxicity was the OAR of most concern. The manual plan's Brain‐PTV V_18Gy_ was 5.69 cc compared to the RapidPlan plan's 5.26 cc, the difference in irradiated normal brain volume as a function of dose is shown in Figure 2. The total PTV received a D_100%_ of 25.6 Gy for the manual plan compared to 25.4 Gy for the RapidPlan plan. There was a decrease in the number of monitor units by 779 MUs from the original plan to the RapidPlan, with the original plan having 2962 MUs and the RapidPlan plan having 2183 MUs. The RapidPlan plan was generated automatically in 15.2 min.

### 3.2 Five‐fraction replanning

Out of the 58 five‐fraction brain SRT plans, seven plans were unable to be used in the DVH estimation tool due to a known memory error within Eclipse v16.1,^25^ heavily associated with the number of metastases. The remaining 51 plans were run through the HARP model, of those 51 plans, 20 plans were identified as clinically acceptable, and the remaining 31 were identified as clinically unacceptable, indicating these plans should not be considered for attempting to reduce the number of fractions. Clinically unacceptable plans were plans that failed the cited OAR criteria. This assessment was often due to OAR proximity and/or the size of the lesions. Of the 31 plans, 23 plans had unacceptable doses to OARs excluding the brain, two plans had unacceptable doses to the brain, and six plans had unacceptable doses to all OARs including the brain. Plan demographics between these groups are shown in Table 4. The unable‐to‐be‐used group is characterized by its high number of metastases with an average lesion per patient of 10.9 ± 2.8 lesions. The unacceptable plans demonstrate a further distance to the isocenter on average, a shorter distance to the nearest OAR, and a larger average PTV volume of 17.73 ± 25.05 cc compared to the acceptable plans’ 5.50 ± 6.87 cc. Future improvements to the model could make some of the unacceptable plans viable, provided a more diverse training dataset could be accrued with greater distances to isocenter and increased OAR proximities. For the replanned acceptable plans, the average number of monitor units was 2847 ± 534 MUs (2229 MUs–4344 MUs), with plans being generated in 12.4 ± 2.6 min (9.0–18.3 min).

**TABLE 4:** Demographics for five‐fraction data outlining the split between plans used, not used, and unable to be used.

		Acceptable	Unacceptable	Unable to be used
Number of plans		20	31	7
Prescription dose	25 Gy	1	4	0
	30 Gy	15	23	7
	35 Gy	4	3	0
	40 Gy	0	1	0
Lesion location	Frontal (L/C/R)	3/1/5	7/3/9	14/3/7
	Parietal (L/C/R)	6/3/4	4/1/5	9/3/9
	Occipital (L/C/R)	3/1/0	2/2/3	4/3/5
	Temporal (L/C/R)	2/1/5	6/3/3	2/1/6
	Cerebellum (L/C/R)	4/1/3	7/7/4	3/4/3
	Other	0	8	0
Total number of lesions		42	74	76
Lesions per patient		2.1 ± 2.0 (1–9)	2.4 ± 1.6 (1–7)	10.9 ± 2.8 (6–14)
Distance to isocenter (cm)		3.73 ± 2.28 (0.02–7.56)	4.01 ± 2.14 (0.00–9.21)	5.78 ± 1.29 (2.31–8.84)
Closest OAR (cm)		2.74 ± 1.69 (0.00–6.55)	2.23 ± 1.83 (0.00–6.71)	3.77 ± 1.69 (0.00–7.46)
PTV volume (cc)		5.50 ± 6.87 (0.21–20.96)	17.73 ± 25.05 (0.22–99.43)	2.59 ± 3.54 (0.06–19.76)
PTV diameter (cm)		2.48 ± 1.29 (0.91–5.46)	3.58 ± 2.20 (0.92–9.58)	1.88 ± 0.97 (0.63–6.70)
Brain volume (cc)		1397 ± 161 (1140–1660)	1340 ± 160 (1107–1732)	1419 ± 100 (1261–1587)

*Note*: When applicable, values are reported as mean ± standard deviation (range). Distance to the isocenter is measured from the isocenter to the center point of the tumor. The closest OAR was measured surface to surface from PTV to the spinal cord, brainstem, or optic pathway. Lesion location reported as left/center/right in the brain parenchyma.

Abbreviations: GTV, gross tumor volume; OAR, organs‐at‐risk; PTV, planning target volume.

#### 3.2.1 Target coverage

**FIGURE 3:**
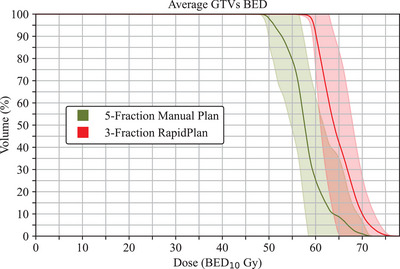
BED volume histograms demonstrating the enhanced BED to the GTVs between the manual and RapidPlan brain SRT plans. The solid lines represent the average GTV value for all 20 five‐fraction HARP plans; the shadowing represents ± 1 standard deviation. BED was calculated with an α/β of 10 Gy. The RapidPlan model achieves at least 5 Gy BED higher than the clinical plans for the majority of the tumor volume.

The 20 five‐fraction brain SRT plans that were replanned to 27 Gy in three fractions were normalized such that all PTVs received at least the prescription dose to 95% of their volume. Target metrics are reported in Table 5. As observed in the three‐fraction testing plans, the PTV D_100%_ is lower than our clinical criteria of 90% of the prescription dose, receiving an average value of 23.5 ± 1.1 Gy. Additionally, the Paddick CI receives an average value of 0.87 ± 0.05, slightly below the 0.90 criteria. However, the plans were deemed clinically acceptable by an attending physician due to the tradeoff of a slightly escalated GTV dose above the criteria, with the D_100%_ receiving 29.1 Gy ± 1.1 Gy. This escalation, along with the change in fractionation scheme, enables the RapidPlan plans to provide a greater BED to the tumor. Using the common standard of α/β = 10 Gy, we find the original manual plans received a BED_10_ D_100%_ of 52.9 ± 4.5 Gy (46.8–64.1 Gy) compared to the RapidPlan plans’ 57.3 ± 3.1 Gy (47.8–61.9 Gy). This difference is highlighted in Figure 3, showing the biologically effective dose volume histograms (BEDVHs) for the average GTVs between both plans.

**TABLE 5:** Target coverage and OAR doses for 20 five‐fraction plans replanned to three‐fractions using the HARP model.

Structure	Metric	SRT protocol criteria	Value
PTV	D_100%_ (Gy)	≥24.3^a^	23.5 ± 1.1 (20.6–25.2)
	Paddick CI	≥0.9^a^	0.87 ± 0.05 (0.65–0.94)
	RTOG CI	1.0–1.5^a^	1.08 ± 0.10 (0.97–1.55)
	HI	≥0.15^a^	0.19 ± 0.04 (0.13–0.28)
	GI	3.0–4.0^a^	3.77 ± 1.27 (1.50–6.64)
GTV	D_100%_ (Gy)	≥27^a^	29.1 ± 1.1 (25.7–30.6)
	D_0.03cc_ (Gy)	<35.1^a^	32.1 ± 1.0 (30.5–34.3)
Brain	V_18Gy_ (cc)	<26^b^	9.9 ± 5.9 (1.3–25.8)
Optic pathway	D_0.2cc_ (Gy)	<13.8^c^	2.0 ± 2.3 (0.4–10.7)
	D_0.03cc_ (Gy)	<17.4^c^	2.8 ± 3.7 (0.4–17.2)
Brainstem	D_0.5cc_ (Gy)	<18^c^	4.9 ± 4.2 (1.0–13.5)
	D_0.03cc_ (Gy)	<23.1^c^	7.6 ± 7.7 (1.1–22.8)
Spinal cord	D_0.35cc_ (Gy)	<18^d^	1.0 ± 0.9 (0.2–4.5)
	D_0.03cc_ (Gy)	<21.9^d^	1.6 ± 2.7 (0.2–13.2)
Skin	D_0.03cc_ (Gy)	<13.5^a^	7.4 ± 2.9 (2.8–12.7)
Left cochlea	D_0.03cc_ (Gy)	<17.1^c^	1.4 ± 1.0 (0.1–3.9)
Right cochlea	D_0.03cc_ (Gy)	<17.1^c^	1.3 ± 0.9 (0.1–3.0)

*Note*: Values are reported as mean ± standard deviation (range). Plans were normalized such that all PTVs’ D_95%_ receive at least the prescription dose.

Abbreviations: GTV, gross tumor volume; PTV, planning target volume.

^a^ Clinical Protocol.

^b^ RSS Guidelines.^24^

^c^ Alliance A071801.^23^

^d^ AAPM Task Group 101.^22^

#### 3.2.2 Dose to OARs

All reported OARs were within the stated criteria (Table 5). In the context of reducing the number of fractions from five to three, a metric of particular interest was the Brain‐PTV V_18Gy_, which received 9.9 ± 5.9 cc for a criteria of less than 26 cc. Although the average values were well below the various criteria, a few of the maximum values were close to the criteria, although still below. For example, the Brain‐PTV received a maximum value of 25.8 cc, 0.2 cc below the threshold, the optic pathway received a value of 17.2 Gy for the max dose, 0.2 Gy under the threshold, and the brainstem received a maximum value of 22.8 Gy, 0.3 Gy under the threshold.

#### 3.2.3 Example case

**FIGURE 4:**
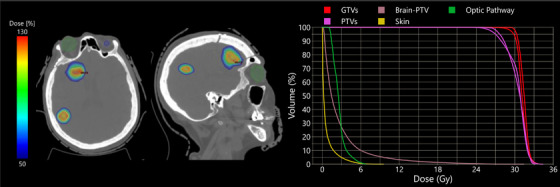
Isodose color wash distributions and DVHs for two brain metastases (1.33 cc and 19.66 cc), originally a five‐fraction brain SRT plan (35 Gy) that was replanned to three‐fractions (27 Gy) using the HARP model. Contours shown on the isodose distributions are the eyes (green/blue), PTVs (pink), and GTVs (red).

Figure 4 shows the isodose distributions and DVHs for one of the five‐fraction SRT replans. This patient had two brain metastases, one located in the right frontal lobe with a PTV volume of 19.66 cc and one located in the right parietal lobe with a PTV volume of 1.33 cc. The patient was originally prescribed 35 Gy in five‐fractions, for the replan using the HARP model, the patient was prescribed 27 Gy in three‐fractions. With the larger than average target volume, the normal brain's V_18Gy_ was 16.06 cc, well below the threshold of 26 cc. Additional OARs of concern were the optic pathway and skin due to the closer proximity of the large metastasis, both structures’ doses were respected. The plan consisted of 2413 MUs and was generated automatically in 10.3 min.

## 4 DISCUSSION

It is important to deliver the most appropriate plan for each patient; this means acceptable OAR doses while achieving adequate target coverage, as well as considering other important aspects of the treatment process, such as time in the clinic. This research demonstrates that RapidPlan combined with HyperArc can provide acceptable high‐quality three‐fraction brain SRT plans for select cases that were previously thought to be only five‐fraction plans, significantly improving clinical workflow and patient comfort and compliance.

The HARP model developed in this research pushed exceptionally hard on reducing the dose to the normal brain tissue and nearby OARs, often at the expense of PTV coverage, in particular PTV D_100%_. When considering the intention of the PTV margins being for setup uncertainty paired with the accuracy achieved by the Encompass patient immobilization system,^26^, ^27^, ^28^ it is understandable to justify the slight decrease in minimum dose to the PTV for an increased BED to the overall tumor provided exceptional patient setup.

It is not the intention of this research to present a technique to be used to replan every and all five‐fraction brain SRT plans created hereon, as demonstrated by the 20 successful plans out of 51 total plans (excluding the seven plans with a TPS error). Instead, this research is meant to provide an alternative option for select patients who could benefit from a shorter overall treatment, provided the various OAR doses and target coverage are deemed clinically acceptable by the physician. Even though a plan might meet all the OAR criteria for a three‐fraction scheme, leaning on the side of caution for near‐threshold values might be the preferred route depending on individual clinical protocols and physician preferences for brain SRT treatments.

Similar studies have shown the effectiveness of RapidPlan models, with few demonstrating the combination between RapidPlan and HyperArc.^19^, ^29^, ^30^, ^31^, ^32^ However, to the best of our knowledge, this is the first report demonstrating the use of a HARP model to reduce the number of fractions necessary for an effective brain SRT treatment. Dumane et al.^31^ demonstrated the efficiency of RapidPlan for a limited number of lesions (≤4) and were unable to combine RapidPlan with HyperArc. Sagawa et al.^32^ developed a HARP model but relied on manual input from the user for their final plans. All plans generated by the HARP model developed in this research were fully automated with no additional user input.

The model developed throughout this research is continuing development as more three‐fraction plans become available to better improve its effectiveness. A more in‐depth analysis of the potential correlations between our metastases’ demographics and the possibility of fraction reduction is warranted and the focus of a future project. Additionally, a single‐fraction model is being developed with the intention of exploring the option of reducing three fractions SRT plans down to a single‐fraction SRS plan for select patients in the future, potentially facilitating same‐day LINAC‐based SRS.

## 5 CONCLUSION

This study generated a HARP model for three‐fraction brain SRT plans and demonstrated the usability of the model to identify and replan select five‐fraction treatments to within clinically acceptable dose criteria for three‐fraction treatments. The brain SRT model provides standardized, high‐quality treatment plans in under 20 min that do not compromise the quality of treatment, delivering acceptable OAR doses, including brain V_18Gy_ with improved BED to tumors. Furthermore, reducing the number of fractions reduces the total treatment time from a course taking 2–3 weeks to a course taking 1–2 weeks for every other day SRT delivery, improving the clinical workflow and providing patients with less time in the hospital and more time with family. This research is currently implemented in our clinic and is being used frequently to generate brain SRT plans for select HyperArc cases. We encourage other clinics to develop their own HARP models or adopt ours to provide physicians with a fast, optimal, and standardized alternative to arduous manual brain SRT planning.

## AUTHOR CONTRIBUTIONS

Damodar Pokhrel and Shane McCarthy conceived the project. Damodar Pokhrel generated all clinical HyperArc plans. Shane McCarthy developed and tested machine learning routines and collected and analyzed data. William St. Clair and Damodar Pokhrel provided their clinical expertise and supervision of the research. Shane McCarthy drafted the preliminary manuscript, and all co‐authors revised and approved the final manuscript for submission.

## CONFLICT OF INTEREST STATEMENT

The authors declare no conflict of interest.

## Appendix

**TABLE A1:** Final optimization objective template used for the three‐fraction HARP model for brain SRT.

Structure	Objective type	Volume	Dose	Priority
GTV	Upper	0.0%	120.0%	115
	Upper	5.0%	117.0%	110
	Lower	100.0%	101.0%	130
	Lower	100.0%	115.0%	100
PTV	Upper	5.0%	110.0%	100
	Lower	100.0%	99.0%	100
	Lower	99.0%	101.0%	130
	Lower	96.0%	103.0%	145
Brain‐PTV	Upper	1.5%	17 Gy	120
	Upper	*	18 Gy	120
	Mean	NA	*	140
	Line (preferring OAR)	*	*	100
Brainstem	Line (preferring OAR)	*	*	75
Cochlea (R/L)	Line (preferring OAR)	*	*	60
Eye (R/L)	Line (preferring OAR)	*	*	60
Lens (R/L)	Line (preferring OAR)	*	*	60
Optic chiasm	Line (preferring OAR)	*	*	70
Optic nerve (R/L)	Line (preferring OAR)	*	*	70
Skin	Upper	0.1%	40%	110
	Line (preferring OAR)	*	*	80
Spinal Cord	Line (preferring OAR)	*	*	70

*Note*: An SRS NTO with a priority of 100 was used. Brain‐PTV is the brain structure minus all PTVs. Values generated by RapidPlan are denoted with an asterisk (*).

Abbreviations: GTV, gross tumor volume; HARP, HyperArc‐based RapidPlan; OAR, organs‐at‐risk; PTV, planning target volume; SRT, stereotactic radiotherapy.
